# From empirical to theoretical models of light response curves - linking photosynthetic and metabolic acclimation

**DOI:** 10.1007/s11120-019-00681-2

**Published:** 2019-10-25

**Authors:** Helena A. Herrmann, Jean-Marc Schwartz, Giles N. Johnson

**Affiliations:** 1 Department of Earth and Environmental Sciences, Faculty of Science and Engineering, University of Manchester, Manchester, M13 9PT UK; 2 Division of Evolution & Genomic Sciences, Faculty of Biology, Medicine and Health, University of Manchester, Manchester, M13 9PT UK

**Keywords:** Photosynthesis, Plant metabolism, Light response curves, Temperature acclimation

## Abstract

Light response curves (LRCs) describe how the rate of photosynthesis varies as a function of light. They provide information on the maximum photosynthetic capacity, quantum yield, light compensation point and leaf radiation use efficiency of leaves. Light response curves are widely used to capture photosynthetic phenotypes in response to changing environmental conditions. However, models describing these are predominantly empirical and do not attempt to explain behaviour at a mechanistic level. Here, we use modelling to understand the metabolic changes required for photosynthetic acclimation to changing environmental conditions. Using a simple kinetic model, we predicted LRCs across the physiological temperature range of *Arabidopsis thaliana* and confirm these using experimental data. We use our validated metabolic model to make novel predictions about the metabolic changes of temperature acclimation. We demonstrate that NADPH utilization are enhanced in warm-acclimated plants, whereas both NADPH and CO_2_ utilization is enhanced in cold-acclimated plants. We demonstrate how different metabolic acclimation strategies may lead to the same photosynthetic response across environmental change. We further identify that certain metabolic acclimation strategies, such as NADPH utilization, are only triggered when plants are moved beyond a threshold high or low temperature.

**Electronic supplementary material:**

The online version of this article (10.1007/s11120-019-00681-2) contains supplementary material, which is available to authorized users.

## Introduction

In order to optimize fitness and yield, plants acclimate to the environmental conditions to which they are exposed. Plant acclimation strategies in response to different environmental conditions include changes in photosynthesis and metabolism (Bassham 1971; Walters 2005; Heyneke and Fernie 2018; Herrmann et al. 2019). Photosynthesis converts inorganic carbon (CO_2_) into organic carbon compounds, using light energy. These organic compounds are either used directly during the day or are stored through the day and utilized at night to support tissue maintenance and biomass production. Dynamic acclimation of photosynthesis (Athanasiou et al. 2010) has previously been shown to alter the metabolic processes which underpin this diel carbon distribution in plant cells (Strand et al. 2003; Dyson et al. 2016; Hurry et al. 2001).

Light response curves (LRCs) describe the relationship between photosynthesis and light intensity. LRCs capture the photosynthetic phenotype of plants: they provide information on the maximum photosynthetic capacity, quantum yield, light compensation point and leaf radiation use efficiency (Akhka et al. 2001; Johnson and Murchie 2011; Lobo et al. 2013). LRCs can therefore be used to capture how photosynthetic phenotypes vary in response to changing environmental conditions. Often, LRCs are described mathematically using the following empirical model (Ögren and Evans 1993; Akhkha et al. 2001), which fits a non-rectangular hyperbola to the measured rates of photosynthesis (*P*; µmol CO_2_ m^−2^ s^−1^) at set irradiances (*I*; µmol photon m^−2^ s^−1^):

1 $$P = \frac{{\varPhi \cdot I + P_{ \hbox{max} } - \sqrt {\left( {\varPhi \cdot I + P_{ \hbox{max} } } \right)^{2} - 4\varTheta \cdot \varPhi \cdot I \cdot P_{ \hbox{max} } } }}{2\varTheta } - R_{\text{d}} ,$$

where *P*_max_ (µmol CO_2_ m^−2^ s^−1^) is the maximum rate of CO_2_ fixation, *Φ* (µmol CO_2_ µmol CO_2_^−1^) the maximum quantum yield of CO_2_ assimilation on an incident light basis, *R*_d_ (µmol CO_2_ m^−2^ s^−1^) the respiration rate measured at 0 µmol photon m^−2^ s^−1^ and *Θ* (dimensionless) determines the curvature of the function. An analogous version of the non-rectangular hyperbola (Eq. 1) is sometimes used to empirically describe the rate of electron transport (often called *J*) for a change in irradiance (Buckley and Diaz-Espejo 2015).

In addition to light, both temperature and the leaf CO_2_ concentration can affect the rate of photosynthesis. Therefore, when describing *P* as a function of *I*, using estimates of *P*_max_, *Θ*, and *Φ*, the temperature and the CO_2_ concentration inside the leaf (which is dependent on both the external CO_2_ concentration and stomatal conductance) should be kept constant and photorespiratory O_2_ fixation should be suppressed. This can be achieved by measuring LRCs under CO_2_-saturating conditions and at a constant temperature. It cannot be excluded that alternative flows to O_2_ (plastid terminal oxidase or Mehler reaction) contribute to the overall electron flux; however, these are generally reported to be low in Arabidopsis and we have not considered them in our analysis (Savitch et al. 2001; Stepien and Johnson 2009).

LRCs have previously been used to quantify photosynthetic acclimation to changing environmental conditions (Savitch et al. 2001; Strand et al. 2003; Dyson et al. 2015, 2016). For example, when plants are grown at high light intensity, they typically have a higher *P*_max_ and a lower *Θ* than plants grown at lower irradiances, reflecting differences in the relative investment in light harvesting and light using structures (Ögren 1993; Evans 1987; Bailey et al. 2001; Athanasiou et al. 2010). Similarly, initial exposure to a drop in temperature slows down metabolism and reduces photosynthetic capacity. This immediate temperature effect on metabolism can be captured using the Arrhenius equation, which describes an exponential relationship between reaction rate and temperature (Arrhenius 1889). This exponential relationship results in a fold increase per linear increase in temperature, most commonly reported for a 10 °C change in temperature, also known as Q_10_. Therefore, as an initial response to cold treatment reactions commonly halve their rate for every 10 °C decrease in temperature, expressed as *Q*_10_ = 2 (Arrhenius 1889; Atkin et al. 2006; Elias et al. 2014). However, dynamic acclimation (acclimation occurring in fully developed mature leaves which is distinct from acclimation during leaf development) to cold of mature plant leaves can then result in a recovery of the photosynthetic capacity, such that the *P*_max_ of cold-acclimated plants equals or surpasses the value of plants in warm conditions (Savitch et al. 2001; Strand et al. 2003; Dyson et al. 2016).

Although LRC parameters other than *P*_max_ change with changing environmental conditions, there are only a handful of examples where this has been explicitly addressed (Krömer et al. 1993; Ögren and Evans 1993; Ögren 1993; Akhkha et al. 2001). Acclimation of these traits may have received little attention thus far because changes in *Φ* are captured only at very low irradiance and changes in *Θ* can be attributed to a wide range of biochemical and structural changes. When *Φ* and *Θ* are high, light capture and utilization are most efficient. Structural changes which may affect photosynthetic efficiency include leaf thickness, light absorption and the composition of the photosynthetic apparatus (Akhkha et al. 2001; Johnson and Murchie 2011). When *Θ* is low, a higher irradiance is required for plants to reach their P_max_. Effectively, this means that more light energy is required for maximum carbon uptake. Therefore, the convexity of LRCs is inversely related to the limitations placed on carboxylation relative to the electron transport (ET) capacity. Under CO_2_-saturating conditions, these limitations may include the supply of NADPH, ATP, ribulose 1,5-bisphosphate carboxylase (Rubisco), cytochrome b_6_f complexes and CO_2_ consumption (Thornley 1976; Marshall and Biscoe 1980; Ögren 1993). While changes in the behaviour of LRCs are frequently attributed to ET rates and flux to assimilated carbon (Krömer et al. 1993; Savitch et al. 2001; Kana and Gilbert 2016), the attributed metabolic changes have not been used to predict LRCs for temperature-acclimated Arabidopsis plants.

Models which link biochemical properties of leaves to gas exchange measurements have been applied for decades (Farquhar et al. 1980; McMurtie and Wang 1993; Long and Bernacchi 2003; Kumarathunge et al. 2019), with the model of C_3_ photosynthesis by Farquhar et al. (1980) being the most commonly adopted. This model considers the kinetic properties of Rubisco, and ribulose 1,5-bisphosphate (RuBP) re-generation, controlled by chloroplast ET, as rate-limiting steps for CO_2_ assimilation in steady state. The model requires species-specific, in vivo, information on the kinetics of carboxylation and oxygenation and the CO_2_ partial pressure in the chloroplast of plants measured. While the Farquhar model and its adaptations have been shown to have great utility when considering carbon assimilation under growth conditions, the model’s accuracy decreases at low and high temperatures (Bernacchi et al. 2001, 2003). While changes in temperature can be modelled using the original model, this temperature dependence describes an immediate temperature effect on plants in their current cellular state (Arrhenius 1889; Elias et al. 2014), but are not sufficient to capture acclimation responses including changes in gene expression and subsequent changes in the proteome and metabolome.

Light response curves can be measured under the conditions in which plants are growing, to provide information about the performance of those plants. In addition, by measuring under a set of constant conditions, information about the *composition* of the photosynthetic apparatus can be obtained. Here, we describe the measurements of LRCs from plants acclimated to a range of temperatures, but measured under a common set of conditions. Fitting these data with an empirical model reveals complex changes resulting from acclimation. To understand better the molecular processes underlying these changes, we have developed a simple metabolic model specific to the assumptions and observations of LRCs measured in CO_2_-saturated conditions. Previous research in our lab has shown that the photosynthetic uptake is the same in control and cold-acclimated plants under growth conditions, but that their LRCs measured in control conditions differ (Dyson et al. 2016). Here, we set out to test whether the observed changes in LRCs can be used to predict the molecular changes required for temperature acclimation and vice versa. For this, we present a metabolic model that links irradiance to carbon assimilation via light-dependent and light-independent reactions using the minimum set of parameters possible. Our validated model allows us to generate predictions about the metabolic changes required for photosynthetic acclimation to changes in temperatures and to further our understanding of the commonly applied empirical model of LRCs (Eq. 1).

## Materials and methods

### Plant material

*Arabidopsis thaliana* (Col-0 accession) plants were grown in three-inch pots, in peat-based compost at 20 °C day/18 °C night, with an 8-h photoperiod and an irradiance of 100 µmol m^−2^ s^−1^ from warm white LED lights (colour temperature 3000–3200 K), as described in Dyson et al. (2016). Plants were kept at a humidity of 68% and bottom-watered three times a week. After 9 weeks, plants were fully grown and subsets of adult plants were transferred 1 h before the onset of the next photoperiod to either 5 °C day/5 °C night, 10 °C day/8 °C night, 15 °C day/13 °C night, 25 °C day/23 °C night, 30 °C day/28 °C night, or kept at 20 °C day/18 °C night temperature conditions for seven days. Temperature treatments are from here on referred to by their day temperature only.

### Light response curves

Light response curves were measured at a temperature of 20 °C using a CIRAS 2 gas analyser (PP systems, Amesbury, USA) fitted with a standard broad leaf chamber. CO_2_ conditions in the chamber were set to 2000 mg L^−1^, at ambient pO_2_ and humidity. Measurements were taken 6 h into the photoperiod after removing plants from their respective cabinets and allowing them to equilibrate to 20 °C conditions for 45 min. Leaves were clamped into the leaf chamber and illuminated using a warm white LED light source at 2000 µmol m^−2^ s^−1^ for 20 min, to reach a steady state of photosynthesis at a relative humidity of 59.46%. They were then exposed for 3–6 min to irradiances of 2000, 1500, 1000, 750, 500, 250, 125 and 0 µmol m^−2^ s^−1^, and the photosynthetic rate (*P*) was recorded once P values were stable for more than 90 s. Gas exchange in the light was corrected for the dark rate, so that only gross photosynthesis is considered. Averages of 3-4 biological replicates for each light curve were taken for each temperature. The data were plotted in R (version 3.4.4) with error bars indicating the standard error across replicates. *P*_max_ measurements taken across the different conditions were tested for significant differences using an unpaired *t* test assuming unequal variances (*p* < 0.05), as implemented in the *stats* package (Version 3.4.4) in R.

### Empirical modelling of LRCs

A non-rectangular hyperbola (Eq. 1) was fitted to the LRC data for each of the six temperatures. Fitting was done using least-squares estimates for non-linear models using the *nls* function in the R *stats* package (Version 3.4.4). Measured values of P for a given I were fitted using the following starting conditions: *P*_max_ = max(*P*) − *R*_d_, *Φ* = 0.08 and *Θ* = 0.9. We normalized LRCs according to the measured *R*_d_ such that *P*(I) = *P*_Measured_(*I*) − *R*_d_. This allowed us to remove the *R*_d_ parameter from Eq. (1) during the model fitting. A Kok effect (Kok 1948, 1949) was not evident at the resolution at which LRCs were measured and was therefore not considered when fitting the empirical model. We plotted both contour and 3-dimensional surface plots for *P* when *P*_max_ ranges from 5 to 10 and *Θ* ranges from 0.1 to 1 for a constant *I* = 100, and *Φ* = 0.027 using the *contour* function in the *graphics* (Version 3.4.4) R package.

### Metabolic modelling

**Fig. 1:**
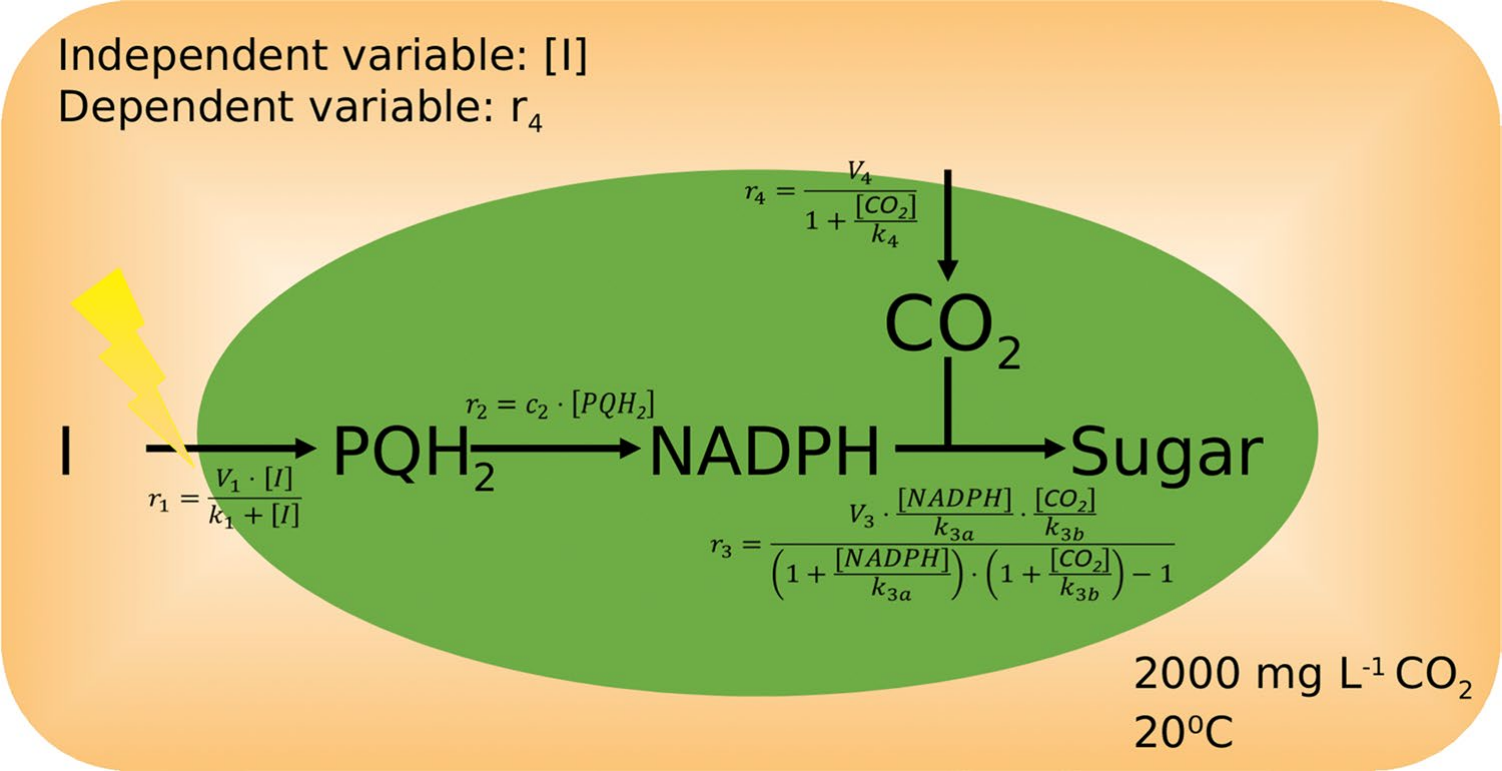
Theoretical model used to capture biochemical changes which link plant CO_2_ assimilation under CO_2_-saturating conditions to changes in light intensity (I). The model captures the formation of nicotinamide adenine dinucleotide phosphate (NADPH) from plastoquinol (PQH_2_), which is used in combination with the assimilated carbon to produce sugars. Rate laws for each of the reactions 1–4 are given. Parameters and their respective units are specified in Table S1. Units area (mol m^−2^) are indicated by square brackets.

A theoretical model linking light intensity to carbon assimilation via simplified light-dependent and light-independent reactions was constructed using the equations and associated rate laws outlined in Fig. 1.

For each independent irradiance [*I*] set in *r*_1_, we associated a dependent rate of photosynthesis (*r*_4_) as measured using the infrared gas analyser. The [CO_2_] concentration is kept at 2000 ppm. Any changes in diffusion through the cell membrane in response to acclimation (e.g. structural changes in the cell membrane) would be captured by a change in *V*_4_ or *k*_4_. The Michaelis–Menten (Michaelis and Menten 1913) reaction r_1_ describes photon capture of the plant, such that *V*_1_ described the maximum light capture. This will depend on the amount of reaction centres present in the plant. The mass action kinetics (Guldberg and Waage 1879) of *r*_2_ describes the amount of electron transport based on the amount of light energy that was absorbed. In combination, *r*_1_ and *r*_2_, describe the electron transport capacity with which photons are turned into high energy molecules (here, collectively referred to as NADPH, since we are assuming linear electron transport). *r*_3_ describes how both an adequate substrate supply of CO_2_ and NADPH in the cell are required for organic carbon production (here collectively referred to as Sugars). This reaction is saturating such that with a surplus of both substrates, the reaction is limited at *V*_3_. Ribulose 1,5-bisphosphate (RuBP) availability, for example could define an upper limit for *V*_3_. *k*_3a_ and *k*_3b_ describe the extent to which internal [CO_2_] and [NADPH] limit sugar production. For example, an inefficiency in Rubisco to bind CO_2_ would increase *k*_3b_. *r*_3_ is represented using convenience kinetics, a generalized form of Michaelis–Menten which makes no assumption on the biding order of multiple substrates (Liebermeister and Klipp 2006). Because the LRCs are measured under CO_2_-saturating conditions, we assumed oxygenation to be negligible. Rather than incorporating all biologically known processes into a single model, we have here opted for a high-level description of the general processes in their simplest form possible. This greatly reduces the number of parameters required, which allowed us to fit the model to our light response curve data without having to imposing any prior assumptions about the model behaviour. The limitation of such a high-level model evidently is that only high-level conclusions can be drawn about the biochemical processes which define temperature acclimation in *A. thaliana*. Given that currently we do not know much about the biochemical process which is regulated in temperature acclimation of *A. thaliana* (Herrmann et al. 2019), this is a good starting point. Model parameters were estimated using the Hooke and Jeeves (1961) parameter estimation algorithm in COPASI (Version 4.24) with an iteration limit of 2000, a tolerance of 10^−10^ and a rho of 0.2. All parameters were estimated from maximum bounds (10^−6^, 10^+6^) with a starting condition of 1. While we cannot be certain about the parameter magnitudes estimated in the following fitting procedure (due to the fact that both light and CO_2_ are model inputs and not outputs), we are able to make general assumptions about the extent to which each parameter defines temperature acclimation in *A. thaliana*. We used LRCs from five temperature conditions (5, 15, 20, 25 and 30 °C) as training data. A useful feature of COPASI is that some parameters can be fitted for all experiments whereas others can be fitted separately for each experiment. We therefore fitted as many parameters as possible across all temperature conditions. We then allowed individual parameters to be estimated separately across temperatures. After testing all possible combinations of parameters which were estimated separately, we chose the minimum possible set that could account for all five temperatures. When allowing *c*_2_ (s^−1^), V_3_ (mol m^−2^ s^−2^), *k*_3a_ (mol m^−2^) and *k*_3b_ (mol m^−2^) to vary across the different temperature sets, we obtained many feasible solutions. Of over 20 possible sets of solutions analysed, we opted for the set of solution which showed the most consistent pattern across temperatures (e.g. *V*_3_ was decreasing with temperature whereas c_2_ was increasing with temperature). The parameter values for this set are summarized in Table S1 along with their respective units. While we acknowledge that other feasible solutions sets exist, we presume that the one which shows the greatest consistency across temperature is likely to be the most biologically relevant.

### Empirical modelling of acclimation

We then plotted the temperature-dependent parameter estimates of the theoretical model and fitted functions (Table S2) to describe their change with temperature using WolframAlpha (Wolfram Research 2019). This allowed us to predict changes in acclimation across temperatures other than those measured. We calculated parameter estimates for *c*_2_, *V*_3_, *k*_3a_ and *k*_3b_ for a temperature of 10 °C and predicted an LRC using the theoretical model.

## Results

### Both *P*_max_ and *Θ* are temperature-sensitive

**Fig. 2:**
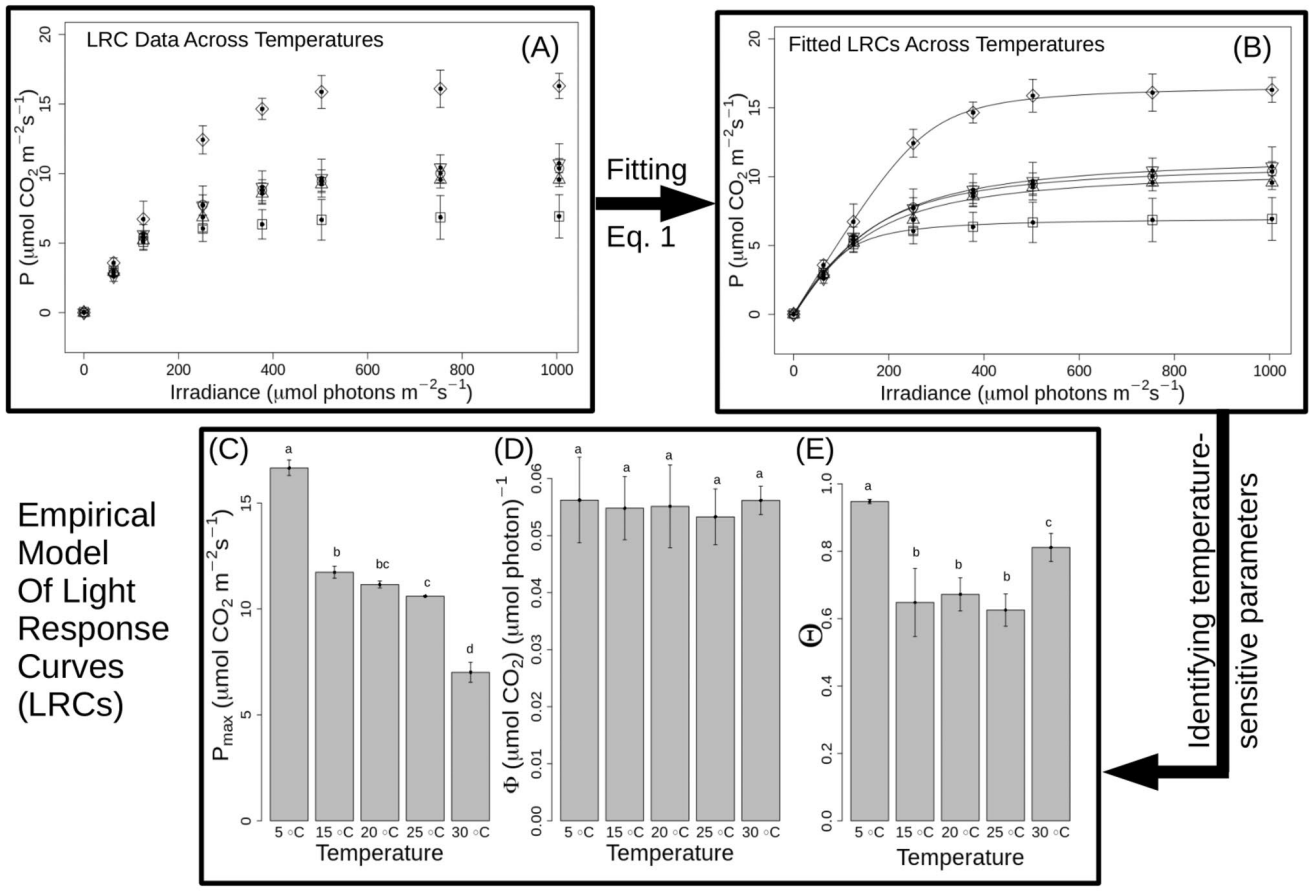
Empirical model of light response curves (LRCs). Using Eq. (1) to fit an empirical model to the photosynthetic rates (*P*) measured across increasing irradiances (I) (**a**, **b**) in order to estimate how the parameters *P*_max_, *Φ* and *Θ* change (**c**–**e**), in plants (*n *= 4) exposed to one week of temperature treatment, after being grown to maturity at 20 °C. Standard mean error (s.m.e.) across the estimates for *P*_max_, *Φ* and *Θ* is shown. Parameter values which are significantly different (*p* < 0.05, unpaired *t* test) are indicated by different letters

Plants of *A. thaliana* grown for 8 weeks at 20 °C were exposed to different temperatures for 7 days and the LRCs of photosynthesis measured under a common set of laboratory conditions (20 °C 2000 mg L^−1^ CO_2_; Fig. 2). Data were then fitted using the empirical model in Eq. (1). Figure 2a and b shows LRCs for control conditions (20°C), two cooler (5 and 15 °C) and two warmer (25 and 30 °C) temperatures which fall within the physiological temperature range of *A. thaliana*. 5 and 30 °C temperature-treated plants demonstrate an acclimation response in *P*_max_ (*p* < 0.05) such that *P*_max_ increases in the cold and decreases in the warm, whereas plants transferred to 15 and 25 °C do not (*p* > 0.05). The observed 5 °C acclimation response confirms existing published data of Col-0 (Dyson et al. 2016). Surprisingly, the *Θ* value of Eq. (1) changes in response to temperature, but such that *Θ* is higher at both low and high temperature (5 and 30 °C). While P_max_ is observed to decrease as a function of temperature (Fig. 2c), *Θ* is observed as a polynomial function of temperature (Fig. 2d). *Φ* does not change with temperature (Fig. 2b). The values of *Φ* estimated by the empirical model are at the lower end of values recorded in other species (Krömer et al. 1993; Björkman and Demmig 1987; Hogewoning et al. 2012); however, caution is needed when comparing values between studies—measurements here are based on incident, rather than absorbed, light. The white LED used here will be differently absorbed to the light sources used elsewhere (e.g. Björkman and Demmig 1987) such that values cannot be directly compared. Nevertheless, all values estimated here are comparable, given that chlorophyll content does not vary significantly in our experiment (Fig. S2). The LRCs and the estimated *P*_max_ and *Θ* values show that there exists a temperature range including 15–25 °C in which no photosynthetic acclimation response is triggered (Fig. 2a, b).

### *P*_max_ and *Θ* define *P* acclimation

**Fig. 3:**
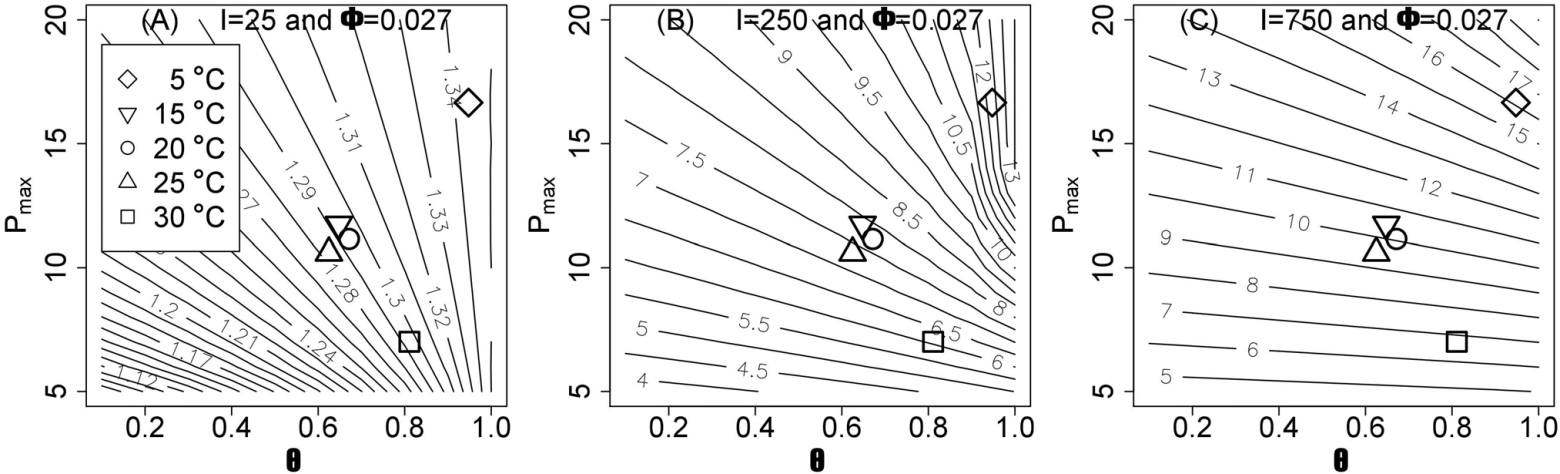
Contour plots showing how *P*_max_ and *Θ* affect *P* (lines) at a given light intensity (**a***I *= 50 µmol m^−2^ s^−1^, **b***I *= 500 µmol m^−2^ s^−1^, **C***I* = 1500 µmol m^−2^ s^−1)^ and for *Φ *= 0.027. Data points shown represent measured data as in Fig. 2, at the light intensities and growth temperatures indicated.

In order to understand the extent to which the observed changes in *P*_max_ and *Θ* with temperature affect *P* in acclimated plants, we drew contour plots of *P* for varying ranges of *P*_max_ and *Θ* (Fig. 3). These demonstrate that different combinations of *P*_max_ and *Θ* can achieve identical values of *P*. At lower light intensities (e.g. 50 µmol m^−2^ s^−1^, Fig. 3a), *Θ* is primarily responsible for a change in P, whereas at higher light intensities (e.g. 1500 µmol m^−2^ s^−1^, Fig. 3c), *P* becomes defined by *P*_max_ only. At intermediate light intensities (e.g. 500 µmol m^−2^ s^−1^, Fig. 3b), both *P*_max_ and *Θ* define the photosynthetic rate. As LRCs are parametrized under CO_2_-saturating conditions and at 20 °C, *P* values do not represent the values observed in growth conditions, but demonstrate the extent of acclimation plants have undergone in response to changes affecting *P*_max_ and *Θ*. For cold-treated plants, the increased *P*_max_ and *Θ* affect *P* across all light intensities. However, the decrease in *P*_max_ observed in response to warm treatment is counteracted by the increase in *Θ* at lower light intensities, such that warm-acclimated plants achieve the same *P* as non-acclimated plants (Fig. 3a). This allows us to conclude that different acclimation strategies can result in the same carbon assimilation. Indeed, plants show similar rates of photosynthesis after 1 week of cold and warm treatment (Fig. S1) even though they obviously have different LRCs when measured in control conditions. This suggests that the plants have acclimated to their new environmental conditions.

### A simple metabolic model allows prediction of LRCs

**Fig. 4:**
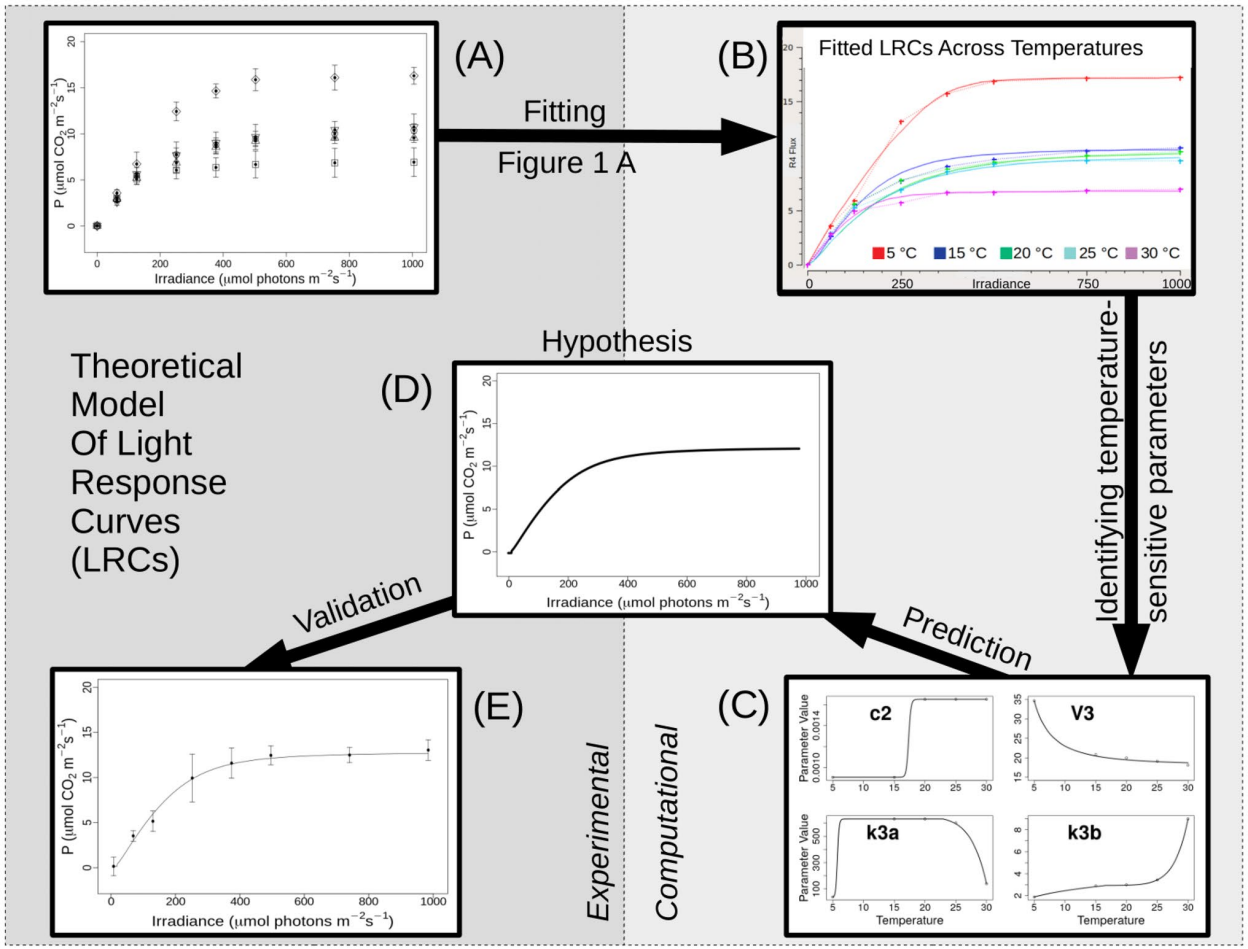
Theoretical model of light response curves. **a**, **b** The equations and rate laws given in Fig. 1 were used to fit a theoretical, metabolic model to the photosynthetic rates (*P*) measured across increasing irradiances (I). The parameter changes required for plants (*n* = 4) exposed to 1 week of temperature treatment, after being grown to maturity at 20 °C, were estimated. **c***c*_2_, *V*_3_, *k*_3a_ and *k*_3b_ were estimated from fitting to the mean data for each given temperature. **d**, **e** After fitting functions through these parameters, the estimated values for 10 °C were used to predict a light response curve (LRC) for that temperature and the model predictions were confirmed experimentally.

While the *P*_max_ parameter of Eq. (1) defines the maximum CO_2_ fixation by plants under saturating light conditions and *Θ* relates to the CO_2_ assimilation on an incident light basis, the biological mechanisms which define *Θ* are less evident. Given that measurements were performed on mature, fully expanded leaves which were temperature-treated for one week only, we assume that no change in leaf thickness, which may affect light absorption, occurred. Furthermore, we confirmed that the total chlorophyll content and the chlorophyll a to b ratio are not affected by the temperature treatment (Fig. S2). The observed changes in response to temperature treatments are therefore likely to be the result of biochemical (e.g. changes in the proteome and metabolome), rather than structural changes (e.g. an increased leaf thickness). In order to explore the biochemical mechanisms which trigger an acclimation response in *P*_max_ and in *Θ* above and below certain thresholds of temperatures, we constructed a metabolic model of LRCs as outlined in Fig. 1. We parametrized this model using the measured LRCs, which allowed us to identify the temperature dependence of specific model parameters (Fig. 4a–c). Our model was able to accurately predict an LRC for 10 °C such that it closely matched the subsequently obtained experimental values (Fig. 4c–e).

### NADPH and CO_2_ utilization change with *P* acclimation

Model construction highlighted a temperature dependence in four parameters which were sufficient to describe the observed changes in LRCs: *c*_2_, *V*_3_, *k*_3a_ and *k*_3b_ (Figs. 1, 4c). c_2_, which defines the conversion of plastoquinol to NADPH, is predicted to be lower in 5–15 °C treated plants than in those exposed to 20–30 °C. *V*_3_, which describes the maximum capacity for the rate of sugar production, is highest at low temperatures and steadily decreases towards 30 °C. *k*_3a_ and *k*_3b_ are binding constants for NADPH and assimilated CO_2_ respectively; *k*_3a_ is lowest at 5 and 30 °C, suggesting a faster NADPH utilization at temperature extremes; *k*_3b_, unsurprisingly, is inversely proportional to *P*_max_.

## Discussion

LRCs have been widely used by plant physiologists for many years to capture the acclimation status of photosynthesis (Ögren and Evans 1993; Leverenz 1988; Thornley 1998; Strand et al. 2003; Johnson and Murchie 2011; Dyson et al. 2016). While it is well known that the relationship between light and photosynthesis reflects underlying physiological and biochemical processes, the most widely used empirical models describing this relationship do not provide any insights into the relative importance of these processes. Here, we have estimated parameters for the empirical non-rectangular hyperbola model of LRCs (Eq. 1). We show that the photosynthetic capacity and curvature parameters shift with temperature acclimation at more extreme temperatures of 5 and 30 °C but are constant across a range from 15 to 25 °C for *A. thaliana* developed at 20 °C. This implies that the processes regulating dynamic photosynthetic acclimation have a degree of buffering capacity, meaning that acclimation only occurs in response to large temperature shifts.

It has previously been shown that temperature acclimation allows plants to maintain a given rate of photosynthesis at different growth temperatures, compensating for changes in enzyme activity by altering enzyme or substrate concentration (Huner et al. 1993; Dewar et al. 2001; Savitch et al. 2001; Dyson et al. 2016). This, however, does not mean that plants which achieve the same rate of photosynthesis at a given light intensity share the same metabolic state. Kitano (2004) defines a robust metabolic system to be one that achieves the same function across different environmental conditions. This function, however, may be achieved in different ways. As outlined above, P is dependent on a range of possible parameter values (*P*_max_, *Θ* and *Φ*). The same value of P can be achieved using different sets of parameter values. This means that the same photosynthetic rate can be achieved across different conditions using different metabolic strategies, which is likely to be the case given that we observe a similar in-cabinet photosynthetic rate but different LRCs across control and acclimated plants. Kumarathunge et al. (2019) analysed a large data set of LRCs of 141 plant species to show that optimal temperatures for photosynthesis are primarily the results of biochemical limitations and that these are largely defined by plant acclimation rather than adaptation. This further demonstrates that plant metabolism has evolved to be robust to environmental change and is likely to consist of a wide range of temperature-specific acclimation strategies.

In order to explore how metabolism changes with temperature acclimation, we constructed a simple kinetic model. While the Farqhuar et al. (1980) model considers up to 42 parameters, at least ten of them are negligible when measuring LRCs under CO_2_-saturating conditions. At least another ten are likely to change as plants acclimate their cellular state to new growth conditions and would lead to a combinatorial explosion of feasible parameter values if we had tried to fit them all as temperature-dependent parameters. For this reason, we chose to design a simple kinetic model relevant only to CO_2_-saturating LRCs measured at a constant temperature in plants acclimated to different growth conditions.

The simplicity of our kinetic model and the fact that we had data from plants acclimated to many temperatures with which to train the model meant that we did not require a detailed prior knowledge of enzyme concentrations or kinetics across different temperatures.

Assuming that, upon 1 week of temperature treatment, plants achieve their new acclimated state (as observed by changes in LRCs) with the minimum metabolic changes possible, we have identified four parameters in our theoretical model which can account for the observed changes in LRCs. These changes cannot be described using Arrhenius law (Arrhenius 1889) as plants are expected to have altered their cellular state as part of the acclimation process (Herrmann et al. 2019) and all LRCs were measured under identical conditions. Our model predicts a slight decrease in ET capacity, modelled by the c_2_ parameter, in response to cold treatment. This may reflect cold-acclimated plants down-regulating their electron transport rate at 20 °C after increasing their ET capacity in response to cold (Price et al. 1995; Kirchhoff et al. 2000; Yamori et al. 2011). The cytochrome b_6_f complex has been suggested as an important site for metabolic flux control (Schöttler and Tóth 2014) and the maximum ET capacity, described as *J*_max_ in Farquhar et al. (1980), has previously been identified as temperature-sensitive (Farquhar et al. 1980). It is therefore not surprising that ET is regulated during the acclimation process. The capacity for using the downstream products from the ET chain, modelled by *V*_3_, is predicted to be highest in cold-acclimated plants. Dyson et al. (2016) showed that *A. thaliana* is able to store more diurnally produced carbon compounds in the leaves in response to cold temperature treatment. If, upon cold acclimation, plants have an increased capacity for storing carbon, they will have an increased capacity for turning over carbon products as they become available in the cell. This is consistent with the observed increase in *V*_3_ in response to cold treatment and may, in part, account for the increased *Θ* observed in the corresponding LRCs. The curvature of an LRC, modelled by *Θ*, occurs at a higher irradiance in cold-acclimated plants which is due to the fact that *Φ* is unchanged. An increased *Θ* may further be explained by an increased affinity for binding NADPH and CO_2_, as indicated by the k_3a_ and k_3b_ constants. Both are lower in cold-acclimated plants which suggest that these plants have acclimated to utilize both carbon and NADPH more efficiently. The two binding constants, k_3a_ and k_3b_, in Reaction 3 (Fig. 1) most closely match the observed changes in *Θ* and *P*_max_, respectively. Plants increase their ability to utilize NADPH in response to both cold and warm temperature extremes; their ability to utilize CO_2_ is only increased in response to cold treatment and decreases at higher temperatures. The *Θ* parameter in the empirical model therefore most closely aligns with the plant’s ability to utilize NADPH in our theoretical model; however, changes in the ET capacity and CO_2_ utilization are also required to further explain the observed acclimation processes (Fig. 1, S1). In combination, these results highlight that no single parameter in our theoretical model that can alone account of the observed changes in LRCs upon temperature acclimation. The fact that the four parameters, *c*_2_, *V*_3_, *k*_3a_ and *k*_3b_, are all required to vary with temperature effectively shows that plant acclimation in response to temperature treatments is defined by changes in the ET chain, the carbon binding affinity and turnover, and NADPH utilization.

The triggers for the above outlined acclimation responses predicted by our model, however, remain unknown. Lack of specificity of biological process which is temperature-sensitive makes it difficult to pin point individual thermosensors (Vu et al. 2019). Considering a system response, as we have done, may thus be more appropriate in order to identify the different coordinating metabolic changes that define the acclimation process. Further detail, however, is required for understanding how the achieved changes in metabolism are obtained.

## Conclusion

Here. we considered photosynthesis as the forefront of metabolic changes in order to study temperature acclimation of *A. thaliana*. We made use of both theoretical and empirical models to capture and understand the metabolic changes required for photosynthetic acclimation. Using a well-established empirical model, we showed that both the capacity and curvature of LRCs vary with temperature acclimation. Using a novel metabolic model, we were able to predict LRCs of *A. thaliana* acclimated to different temperature conditions for 1 week. We were able to validate our model prediction for plants acclimated to 10 °C experimentally. We made predictions on the biochemical changes that are required for the observed changes in LRCs in response to temperature treatment. Our theoretical model confirms known coordinated changes in ET and carbon utilization in response to temperature, and highlights NADPH utilization as a potential proxy for the curvature of LRCs measured under CO_2_-saturating conditions. Future work which addresses how the temperature conditions of plant development impact the range of temperatures that trigger an acclimation response in adult plants may shed further light on the underlying mechanism that are triggered for a metabolic and photosynthetic acclimation response.

## Electronic supplementary material

Below is the link to the electronic supplementary material.

Supplementary material 1 (DOCX 38 kb)
