# Supplementary material for: From empirical to theoretical models of light response curves - linking photosynthetic and metabolic acclimation
Source: Photosynth Res. 2019 Oct 25;145(1):5–14. doi: 10.1007/s11120-019-00681-2 (PMC7308256; doi:10.1007/s11120-019-00681-2)
Supplement: Supplementary file 1 — Supplementary material 1 (DOCX 38 kb) [file 11120_2019_681_MOESM1_ESM.docx]

**Supplementary Information**

**Table S1** Parameter estimates obtained when fitting the theoretical model to the observed light response curves (LRCs).

| **Parameter** | **Units** | **Experiment** | **Value** |
| --- | --- | --- | --- |
| V_1_ | mol m^-2^ s^-1^ | 5, 15, 20, 25, 30 °C | 34.00 |
| k_1_ | mol m^-2^ | 5, 15, 20, 25, 30 °C | 3.8e-6 |
| c_2_ | s^-1^ | 5, 15 °C | 0.00091 |
| c_2_ | s^-1^ | 20, 25, 30 °C | 0.00165 |
| V_3_ | mol m^-2^ s^-1^ | 5 °C | 34.6 |
| V_3_ | mol m^-2^ s^-1^ | 15 °C | 21.04 |
| V_3_ | mol m^-2^ s^-1^ | 20 °C | 20.07 |
| V_3_ | mol m^-2^ s^-1^ | 25 °C | 19.13 |
| V_3_ | mol m^-2^ s^-1^ | 30 °C | 17.71 |
| k_3a_ | mol m^-2^ | 5 °C | 40.027 |
| k_3a_ | mol m^-2^ | 15, 20 | 631.02 |
| k_3a_ | mol m^-2^ | 25 °C | 601.851 |
| k_3a_ | mol m^-2^ | 30 °C | 140.129 |
| k_3b_ | mol m^-2^ | 5 °C | 1.883e-5 |
| k_3b_ | mol m^-2^ | 15 °C | 2.899e-5 |
| k_3b_ | mol m^-2^ | 20 °C | 3.000e-5 |
| k_3b_ | mol m^-2^ | 25 °C | 3.197e-5 |
| k_3b_ | mol m^-2^ | 30 °C | 8.979e-5 |
| V_4_ | mol m^-2^ s^-1^ | 5, 15, 20, 25, 30 °C | 75.8 |
| k_4_ | mol m^-2^ | 5, 15, 20, 25, 30 °C | 5.47e-6 |

**Table S2** Equations used to describe the temperature-dependence of the parameters which were identified to be temperature sensitive

| **Parameter** | **Units** | **Equation** |
| --- | --- | --- |
| c_2_ | s^-1^ | 0.00165+(0.000907-0.00165)/(1+(x/17.33976)^90.23686) |
| V_3_ | mol m^-2^ s^-1^ | 17.946657+(11570010-17.94657)/(1+(x/0.001928826)^1.71139) |
| k_3a_ | mol m^-2^ | For x<23:  631+(38.83458-631)/(1+(x/5.763419)^43.93565) For x>=23: -101800700+(651.2443+101800700)/(1+(x/82.81671)^12.01704) |
| k_3b_ | mol m^-2^ | For x<16.5: 432.1053+(-0.7567627-432.1053)/(1+(x/411790700)^0.2794011) For x>=16.5: 525603.4+(2.936102-525603.4)/(1+(x/68.80208)^13.70013) |

**
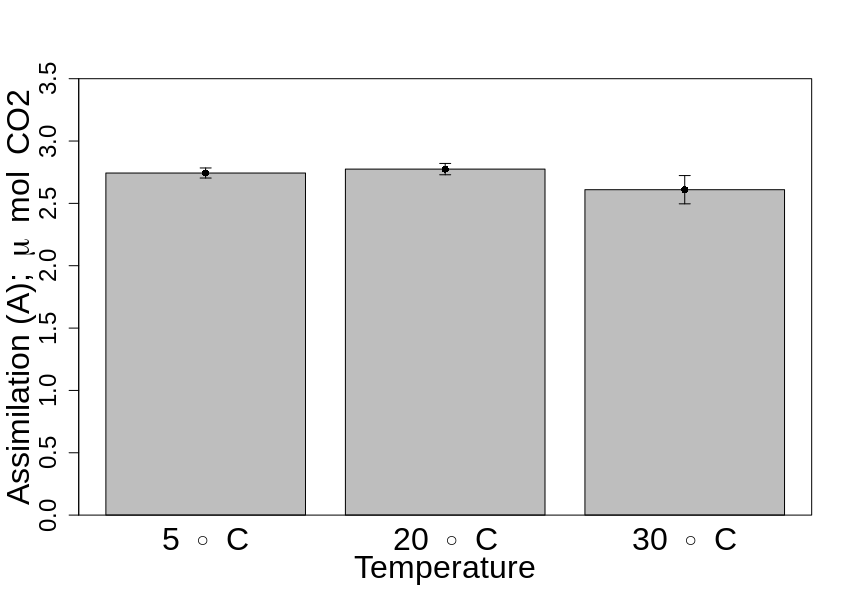
**

**Fig S1** In cabinet measurements of photosynthesis taken in between the 5th-6th hour of the photoperiod of control plants and plants which have been treated with one week of 5 °C or one week of 30 °C. The mean standard errors of 3 replicates are shown.

**
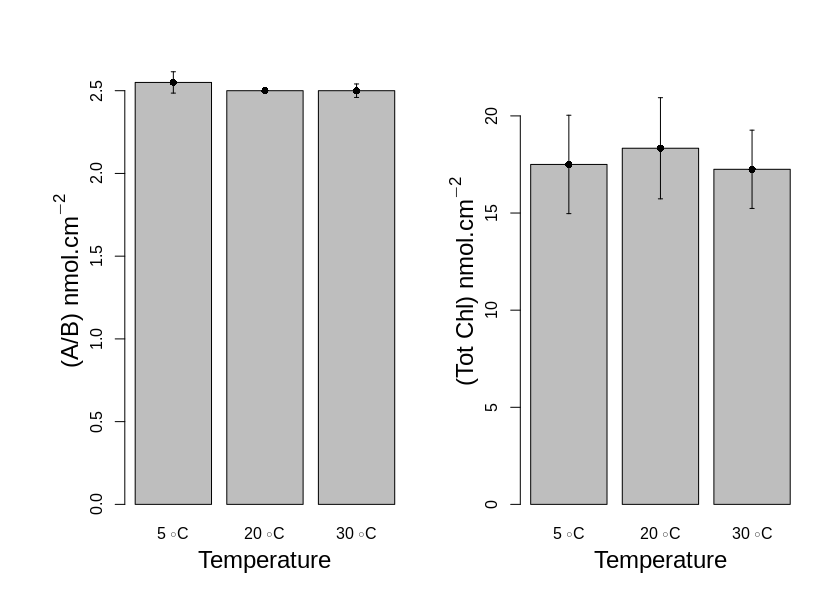
Fig S2** Chlorophyll a:b ratio (left) and total chlorophyll (right) content per leaf area (cm^2^). Error bars indicate the standard error of 3-4 replicates for each temperature. Chlorophyll content was estimated in 80% v/v acetone, using the method of Porra et. al. (1989)

**Supplementary references:**

Porra P., Thompson W.A. and Kriedmann P.E. (1989) “Determination of accurate extinction coefficients and simultaneous equations for assaying chlorophylls a and b extracted with four different solvents: verification of the concentration of chlorophyll standards by atomic absorption spectroscopy” Biochim Biophys Acta, 975, 384-394
